# RETRACTED: Cejuela et al. Metformin and Breast Cancer: Where Are We Now? *Int. J. Mol. Sci.* 2022, *23*, 2705

**DOI:** 10.3390/ijms26115407

**Published:** 2025-06-05

**Authors:** Mónica Cejuela, Begoña Martin-Castillo, Javier A. Menendez, Sonia Pernas

**Affiliations:** 1 Department of Medical Oncology, Catalan Institute of Oncology (ICO)–L’Hospitalet de Llobregat, 08908 Barcelona, Spain; monicacejuela@iconcologia.net; 2 Unit of Clinical Research, Catalan Institute of Oncology (ICO)–Girona, 17007 Girona, Spain; bmartin@iconcologia.net; 3 Girona Biomedical Research Institute (IDIBGI), Salt, 17190 Girona, Spain; jmenendez@idibgi.org; 4 Program Against Cancer Therapeutic Resistance (ProCURE), Metabolism and Cancer Group, Catalan Institute of Oncology (ICO)–Girona, Salt, 17190 Girona, Spain; 5 Breast Cancer Group, Institut d’Investigació Biomèdica de Bellvitge–IDIBELL, L’Hospitalet de Llobregat, 08908 Barcelona, Spain

The journal retracts the article titled “Metformin and Breast Cancer: Where Are We Now?” [1], cited above. 

Following publication, concerns were brought to the attention of the Editorial Office regarding the unauthorized use of propriety data presented within this article [1]. 

Adhering to our complaints procedure, the Editorial Office and Editorial Board conducted an investigation that confirmed that appropriate permission to publish the underlying data presented in this article had not been obtained. As retroactive permission could not be acquired, the Editorial Board has decided to retract this article as per MDPI’s retraction policy (https://www.mdpi.com/ethics#_bookmark30). 

This retraction was approved by the Editor-in-Chief of the *International Journal of Molecular Sciences*. 

The authors disagreed with this decision.
